# Learning Environmental Field Exploration with Computationally Constrained Underwater Robots: Gaussian Processes Meet Stochastic Optimal Control

**DOI:** 10.3390/s19092094

**Published:** 2019-05-06

**Authors:** Daniel Andre Duecker, Andreas Rene Geist, Edwin Kreuzer, Eugen Solowjow

**Affiliations:** 1 Institute of Mechanics and Ocean Engineering, Hamburg University of Technology, 21073 Hamburg, Germany; geist@is.mpg.de (A.R.G.); kreuzer@tuhh.de (E.K.); 2 Max Planck Institute for Intelligent Systems, 70569 Stuttgart, Germany; 3 Siemens Corporate Technology, Berkeley, CA 94704, USA; eugen.solowjow@siemens.com

**Keywords:** autonomous exploration, environmental field monitoring, gaussian processes, gaussian markov random fields, kalman filtering, stochastic optimal control

## Abstract

Autonomous exploration of environmental fields is one of the most promising tasks to be performed by fleets of mobile underwater robots. The goal is to maximize the information gain during the exploration process by integrating an information-metric into the path-planning and control step. Therefore, the system maintains an internal belief representation of the environmental field which incorporates previously collected measurements from the real field. In contrast to surface robots, mobile underwater systems are forced to run all computations on-board due to the limited communication bandwidth in underwater domains. Thus, reducing the computational cost of field exploration algorithms constitutes a key challenge for in-field implementations on micro underwater robot teams. In this work, we present a computationally efficient exploration algorithm which utilizes field belief models based on Gaussian Processes, such as Gaussian Markov random fields or Kalman regression, to enable field estimation with constant computational cost over time. We extend the belief models by the use of weighted shape functions to directly incorporate spatially continuous field observations. The developed belief models function as information-theoretic value functions to enable path planning through stochastic optimal control with path integrals. We demonstrate the efficiency of our exploration algorithm in a series of simulations including the case of a stationary spatio-temporal field.

## 1. Introduction

**Figure 1:**
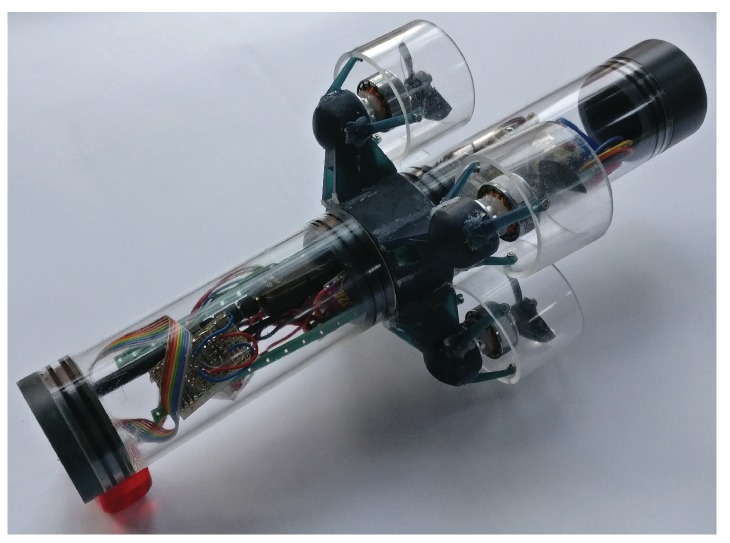
Micro underwater robot HippoCampus for environmental field exploration.

Autonomous underwater field exploration has been a fast-growing research area in the last decade. With continuous advances in small-scale computing technology, smart micro-robots are expected to play a prominent role in increasingly autonomous and interconnected exploration and monitoring systems [1]; examples of micro underwater robots include the Avexis [2] and the HippoCampus [3] micro autonomous underwater vehicle, see Figure 1. Autonomous multi-agent swarms can be deployed for maritime exploration tasks such as the monitoring of algae growth, oil spill, or underwater currents.

Hereby, the development of maritime exploration and monitoring systems profits from many synergies with research on similar onshore tasks such as the monitoring of urban environmental fields. As a result, micro-robots can monitor environmental processes such as particulate matter, acoustic pollution, or electromagnetic fields. Particularly in hazardous environments, autonomous micro-robots inherit great potential to drastically increase safety and reduce costs compared to concepts involving the deployment of stationary sensors or humans taking measurements. Thereby, depending on the dynamics of the specific robotic platform sophisticated control algorithms such as [4,5] are required in order to allow a safe and robust deployment in the underwater domain. However, missions with mobile underwater robots inherit the challenge of limited communication bandwidth and range. This naturally enforces a high level of autonomy as all key components of the exploration algorithm have to run onboard.

**Figure 2:**
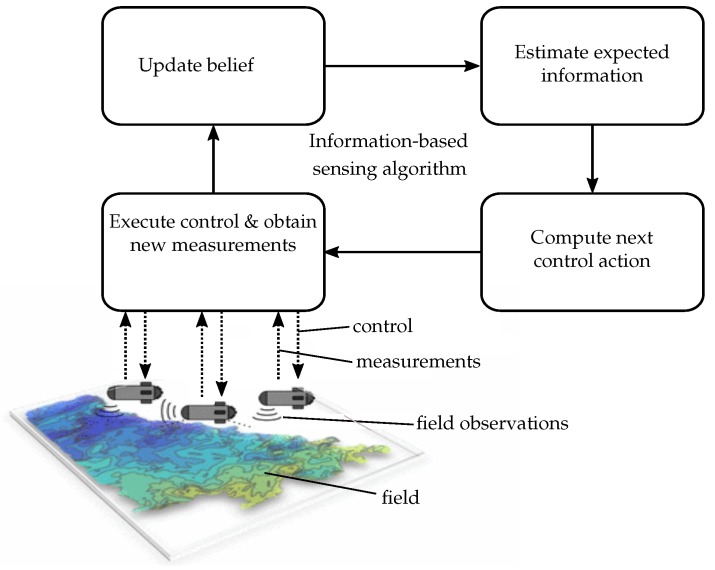
Components of a closed-loop, information-based sensing algorithm for field exploration with autonomous underwater robots.

Systems for maritime exploration tasks consist of multiple building blocks as depicted in Figure 2. The state of the environment is represented in a so-called belief model which is updated through measurements of the environment. The observations are collected using mobile robots. Thus, a path-planning algorithm is required in order to maximize the expected information gain through future observations along the robots’ paths. Since the robots cannot simply take measurements at arbitrary locations, a trade-off arises between the potential information gained and the effort to drive the robots to regions where information-rich observations can be collected.

### 1.1. Related Work

Methods for modeling a robot’s environmental belief can be distinguished in physics-based models and non-physics based models. Physics-based models allow the extrapolation of the model to the vicinity of the known belief. Nonetheless, they require solving a partial differential equation (e.g., Navier–Stokes equation), which is computationally demanding. Moreover, such systems require information regarding the boundary conditions which is often not directly available to the system [6]. In recent years, probabilistic belief models have been developed as a promising alternative for efficient field estimation. These data-driven models may be more suitable for computationally constrained robot swarms since they avoid computational-costly solving the partial differential equation and additionally allow to model the uncertainty of the estimated physical process directly. Moreover, data-driven models inherit the potential to infer the process characteristics during operation.

A prominent inference method for learning environmental field beliefs is Gaussian process (GP) regression. Gaussian process regression, or in geo-statistics terminology ’Kriging’, originated as a method for statistical inference of ore concentration fields [7]. Modeling environmental fields with GPs is attractive as their mean and covariance functions allow a spatial continuous field representation while additionally providing statistical uncertainty measures. Example applications include the estimation of a temperature field in lakes [8] and the reconstruction of spatio-temporal fields with mobile sensor nodes [9]. Moreover, Xu et al. [10] present a broad variety of different GP models for spatial field estimation.

While the continuous specification of a GP provides an intuitive interpretation of the underlying physical process, real-world applications of GPs are often hindered by the big *n* problem. The big *n* problem of GPs is particularly problematic when dealing with multidimensional dynamical processes. Although several methods have been proposed to overcome this constraint, e.g., by reducing the dimensions, simplifying the structure of the covariance matrix [11], or truncating the observations [9], these methods inevitably inherit the trade-off between approximation quality and computational cost. The computational burden of GPs becomes especially challenging and often intractable when resource-constraint underwater robots are involved. In contrast to surface scenarios, high-bandwidth communication links are often not available in the submerged domain. The low-bandwidth communication requires the mobile robots to maintain a decentralized field belief representation on-board. Recently, it has been shown that GPs can be sufficiently approximated through Gaussian Markov random fields (GMRFs). A GMRF approximates a GP on a predefined lattice of random variables (RV) by utilizing the Markov property [12]. Xu et al. discussed in a series of publications the suitability of GMRFs for mobile sensor networks [10,13]. In their recent work, Jadaliha et al. [14] present an extended GMRF framework for mobile robots which incorporates uncertainties in the observation location. In an alternative approach for circumventing the big *n* problem, Todescato et al. [15] combine Kalman filtering techniques with GPs to update a spatio-temporal field representation.

Regarding the task of efficient information gathering, planning algorithms can be grouped into four categories. *Myopic planning* algorithms are computationally efficient as they compute the next best action without a planning horizon. However, they suffer from the risk of getting stuck in local optima. *Sampling-based* strategies have gained increasing interest in recent years, but do not provide guarantees on global optimality; examples include the popular rapidly exploring random tree methods. *Dynamic programming* is the method of choice for informative path planning tasks which can be formulated as a (partially observable) Markov decision problem. However, they are often intractable if the exploration task shall be solved without a state and domain discretization. In their work [16] Hollinger and Sukhatme examine sampling-based information-gathering algorithms for continuous space. They include information quality metrics and motions constraints in their planning algorithms. Marchant and Ramos [17] present a double layered informative path planning concept using GPs and Bayesian optimization, whereby they use one GP to model the physical phenomenon and a second GP to model the quality of the selected paths. The resulting paths are described through cubic splines. In [18], the exploration problem is formulated as a POMDP. The authors use Monte-Carlo tree search and an upper confidence bound for trees together with sequential Bayesian optimization techniques. They evaluate their method in a series of experiments to analyze the performance. Recently, Cui et al. proposed the combination of mutual information and rapidly-exploring random trees for underwater path planning in a scalar field sampling scenario [19].

In this context, many publications consider the scenario of exploring large-scale environments such as oceans. However, when reducing the scale to confined environments such as industrial tanks, the robot dynamics cannot be neglected anymore. This problem can be tackled using *receding horizon* schemes that optimize the full planning horizon while not providing guarantees beyond the horizon. However, such a planning algorithm require a continuous representation of the field belief. Kreuzer and Solowjow use linear functions to interpolate the field belief representation between the grid-points while Xu et al. [20] propose sinusoidal weighting functions which are an attractive nonlinear alternative for smooth field interpolation. The computational costs of these belief algorithms increase with the number of collected observations, rendering their application on real robots impractical. With a continuous belief representation, an exploratory path can be computed using the policy improvement with path integrals (PI${}^{2}$) algorithm proposed by [21]. The PI${}^{2}$ algorithm origins from the work on solving a nonlinear stochastic optimal control via the use of path integrals [22]. In reinforcement learning terminology, our resulting exploration method could be seen as performing policy iteration. In which, first, the exploration policy is evaluated at each new location through an update of the field belief model. Subsequently, the belief is used as an information-theoretic value function for policy improvement as proposed by [23]. This approach enables rapid evaluation of the belief space for finding an optimal informative path.

### 1.2. Contributions

The contribution of this work is two-fold. First, we combine and extend approaches from previous works [13,23] on field exploration with GMRFs to meet the requirements of an application on a micro-robot platform and on the PI${}^{2}$ stochastic path planning algorithm. These requirements are

- a constant computational complexity over time,
- a continuous spatial belief representation which allows efficient path planning.

Therefore, we first extend the PI-GMRF approach proposed in [23] with the concept of sequential Bayesian spatial prediction in [13] to guarantee a constant computational load while maintaining the ability to incorporate off-grid measurements. Second, we extend the recently presented spacetime Kalman filter (STKF) [15] by incorporating the concept of weighted shape functions to render the belief model compatible with PI${}^{2}$. The combination of these two different belief models with PI${}^{2}$ results in two novel algorithms for stochastic field exploration—namely, an improved PI-GMRF and the new PI-STKF.

We conduct two numerical experiments to compare these models regarding their ability to efficiently explore environmental fields and computational complexity. We assume that sufficient prior knowledge on the physical process is available and thus *no hyperparameter estimation* of the belief model is required. For the sake of brevity, we present the field estimation schemes for a *single robot*. Albeit, we derive the algorithms in a form that allows a direct extension to multiple agents sharing a centralized belief model.

### 1.3. Paper Structure

The paper is structured as follows. In Section 2, we briefly outline the problem of autonomous field exploration. In Section 3, the belief models, namely the fully Bayesian GMRF and STKF, are introduced and extended to incorporate off-grid measurements. In Section 4, we elaborate on the usage of PI${}^{2}$ for path planning using information-theoretic value functions. In Section 5, the two belief models are compared with respect to their computational time and estimation results. In Section 6, we analyze the exploration performance of the final algorithm on an unknown spatio-temporal field. Finally, Section 7 concludes this work and highlights potential future research directions.

## 2. Problem Statement

We consider an autonomous underwater vehicle which explores an environmental field through point-wise observations in a confined environment. The goal is to minimize the error between the estimated and the true field within a minimal amount of time. Regarding the exploration algorithm, this task can be restated as maximizing the ratio of collected information per time step. To make this task tractable for mobile robot teams, the computational cost of the exploration algorithm has to be bounded over time.

### 2.1. Robot Model and Problem Formulation

A dynamical robot model allows sampling feasible exploratory trajectories which can be evaluated regarding their potential information gain on the environmental field belief. The motion of each robot is described by

(1) $${\dot{x}}_{t}=f{}_{\mathrm{robot}}\left(x_{t}\right)+G\left(x_{t}\right)u_{t},$$

where $x_{t}\in R^{n}$ is the state of the robot. The passive dynamics $f{}_{\mathrm{robot}}$ define the state transition, which for path planning algorithms is commonly described through a simple particle model. The scaled control input $u_{t}\in R^{p}$ is the computed state correction through the robot’s actuators and $G\left(x_{t}\right)\in R^{n\times p}$ the control matrix.

At each discrete time step, the robot collects measurements $y(x_{t})$ to gather information about the environmental field $f(x_{t})$ of interest. The sensor model of a single robot is described by

(2) $$y\left(x_{t}\right)∼h\left(f(x_{t}),v\right),\;\mathrm{with}\;v∼N\left(0,\Sigma_{z}\right),$$

where $z_{\mathrm{real}}$ depicts the real environmental field values, h(·) is the observation function, and $\Sigma_{y}$ is the diagonal covariance matrix representing the measurement noise.

### 2.2. Field Belief Representation

In order to efficiently learn an environmental field estimate the robots have to gather information by taking measurements and infer knowledge from the collected data. The collected data is mapped to a belief of the underlying environmental process that enables the agents to plan actions. In the information-theoretic exploration algorithms, the second order moments of the field belief representation are used to evaluate the quality of the planned exploration paths. In this context, the concept of probabilistic kernel models is a natural choice for spatially correlated field values. The correlation of the field values is assumed to be known a priori. Thus, no hyperparameter estimation has to be performed during exploration.

The limited computational capacities of embedded systems such as mobile robots require the belief model to have a comparably small and constant computational cost. Therefore, we extend algorithms based on GMRFs and Kalman Filtering to limit their computational complexity and to enable their combination with a stochastic path planning controller for exploration tasks.

A GMRF defines a (finite-dimensional) random field that follows a multivariate Gaussian distribution while satisfying the Markov property. Due to the Markov assumption, the inverse of the covariance matrix Λ can be defined on a predefined lattice, while also being (desirably) sparsely populated. The GMRF is fully defined by a mean vector μ and a precision matrix $\Lambda^{-1}$ as $N(\mu ,\Lambda^{-1})$. GMRFs are well suited for the approximation of conditional auto-regressive processes, but require the initialization of a fixed lattice of random variables (RVs). The initialization hinders the application of GMRFs to model temporal process correlations. Therefore, a Kalman regression algorithm, also referred to as spacetime Kalman Filtering (STKF), is utilized to express a belief of the spatio-temporal environmental field. However, both stochastic field belief models provide a measure of belief uncertainty in the form of the conditional variance.

### 2.3. Stochastic Optimal Control Problem

The stochastic optimal controller allows computing an optimal path with respect to the given value function. In this work, we use a field uncertainty based value function, whereby the field uncertainty is computed from the previously developed belief model. We use a stochastic optimal controller to plan a maximal informative path with respect to the belief model’s predicted variance. As thoroughly discussed in [24], the predicted variance results in the conditional entropy, which is an indirect informative criterion. Hereby, the term indirect informative means that only the uncertainty of the next potential state is considered, while the information of surrounding field values is not considered. A critical insight for information theoretic path planning is that the posterior variance does not depend on the process values of the obtained measurements if the kernel function is known.

In order to sample potentially feasible paths, the following robot dynamics are considered

(3) $${\dot{x}}_{t}=f{}_{\mathrm{robot}}\left(x_{t}\right)+G\left(x_{t}\right)\left(u_{t}+\varepsilon_{t}\right),$$

where $x_{t}\in R^{n\times 1}$ denotes the system state, $f{}_{\mathrm{robot}}\left(x_{t}\right)\in R^{n\times 1}$ the passive dynamics and $\varepsilon_{t}\in R^{p\times 1}$ the additive Gaussian noise with variance $\Sigma_{\varepsilon }$. Moreover, let the index *t* denote any arbitrary time step, while we use the index $t_{i}$ to emphasize a particular time. The final goal of a stochastic optimal controller is to compute the optimal controls $u_{t}$ with respect to the performance functional

(4) $$V\left(x_{t_{i}}\right)=V_{t_{i}}=\min_{u_{i:(H-1)}}E_{\tau_{i:H}}\left(R(\tau_{i:H})\right).$$

The expectation $E_{\tau_{i:H}}$ is taken over all trajectories starting at $x_{t_{i}}$. Also $t_{0}=0\;s$ denotes the time at the current agent position, and $t_{H}$ the last time step of the control horizon. We define the finite horizon cost function $R_{\tau_{i:H}}$ for a trajectory piece $\tau_{i:H}$ with start at time $t_{i}$ and end at $t_{H}$ as

(5) $$R_{\tau_{i:H}}=\phi_{t_{H}}+\int_{t_{i}}^{t_{H}}r_{t}\;dt.$$

The term $\phi_{t_{H}}$ denotes a terminal reward at time $t_{H}$. The immediate cost $r_{t}$ at time *t* is chosen as

(6) $$r_{t}=r\left(x_{t},u_{t},t\right)=q_{t}+\frac{1}{2}u_{t}^{⊤}Ru_{t},$$

with $q_{t}=q\left(x_{t},t\right)$ being a state-dependent cost function, and R being a positive semi-definite weight matrix. Note that the control action $u_{t}$ is linear in (3) and quadratic in (6).

## 3. Probabilistic Belief Modeling for Field Exploration

In this section, we present extensions to two existing field belief concepts, namely the GMRF and the STKF approach, to allow information-based exploration control with constant computational cost over time. Both belief algorithm can be used to estimate a stochastic process on a predefined lattice of Gaussian random variables.

In order to enable the incorporation of the belief models into a stochastic optimal control exploration framework, we extend the belief algorithms analog to [23]. Therefore, we incorporate off-grid observations through an affine transformation of field measurements onto the belief grid using spatial shape functions. However, the original concept presented in [23] does not fulfill the requirement of constant computational cost over time.

The GMRF-based belief algorithm was originally proposed in [13] and enables efficient estimation of stationary spatial processes on a discrete grid of Gaussian random variables. The second belief algorithm, proposed in [15], combines GP regression of spatial process components with Kalman filtering of conditional-auto-regressive temporal process components.

Defining the field representation on a lattice raises the question on how to choose the grid discretization based on the fundamental trade-off between the accuracy of the field representation versus the available computational power. The latter aspect is of particular importance in the field of underwater robotics, as off-board computation of the field representation is not feasible due to the very limited communication bandwidth. Thus, the discretization step size has to be selected depending on the actual application scenario. Thereby, prior knowledge on the spatial scale of the physical process is a helpful and valid assumption. For instance, if the user aims to explore small scale processes in a local environment, e.g., an industrial tank, a dense grid is likely to be a better choice than a coarse grid which captures global physical phenomena with acceptable computational burden. Moreover, shape function approximation can be used to interpolate the field belief between the grid points. This allows to efficiently monitor large scale fields where the main interest lies in the exploration of global phenomena rather than local small scale processes.

### 3.1. Shape Functions

**Figure 3:**
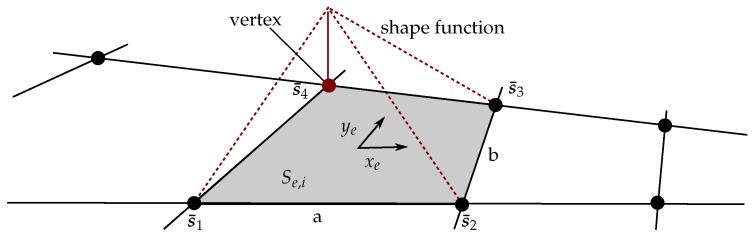
Shape function on a selected grid element.

The introduced belief algorithms estimate the field on a discrete lattice {V,E} with vertices V={1,…,n} and edges E. The set of continuous field locations is discretized into a finite subset of *n* spatial input locations $S=\{s_{1},\ldots ,s_{n}\}$, such that the vector  $f\left(t\right)={\left[f\left(s_{1},t\right),\ldots ,f\left(s_{n},t\right)\right]}^{⊤}≜{\left[f_{1,t},\ldots ,f_{n,t}\right]}^{⊤}$ is a discretization of f(x,t). The lattice S consists of a finite number of sub-domains $S_{e,i}$, where each is enclosed by four vertices ${\bar{s}}_{i}$, with i=1,…,4. For the ease of illustration, S is chosen as regular lattice with edges each of length *a* and *b* respectively, as depicted in Figure 3. As proposed by [23], the field value at position q can be approximated through a sum of weighted shape functions $\phi_{i}^{e}\left(q\right)$ and field values on the vertices $f({\bar{s}}_{i})$, such that

(7) $$F^{e}=\sum_{i=1}^{4}\phi_{i}^{e}\left(q\right)f\left({\bar{s}}_{i}\right)$$

Figure 3 illustrates shape function $\phi_{i=4}^{e}$ on an element domain $F_{e}$. Each shape function is zero outside its corresponding element and equal to one at the associated vertex

(8) $$\phi_{i}^{e}\left({\bar{s}}_{k}\right)=\left\{\begin{array}{cc} 1, & \mathrm{if}\;i=k\\ 0, & \mathrm{otherwise} \end{array}\right..$$

A local coordinate system $K_{e}\left(x_{e},y_{e}\right)$ is defined on $F_{e}$. The origin of $K_{e}\left(x_{e},y_{e}\right)$ lies in the center of the element. The corresponding coordinate axis are orthogonal to each other and parallel to the respective element edges. The shape functions are defined as

(9) $$\begin{array}{c} \begin{array}{cccc} \phi_{1}^{e} & =\frac{1}{ab}\left(x_{e}-\frac{a}{2}\right)\left(y_{e}-\frac{b}{2}\right), & \phi_{2}^{e} & =-\frac{1}{ab}\left(x_{e}+\frac{a}{2}\right)\left(y_{e}-\frac{b}{2}\right),\\ \phi_{3}^{e} & =\frac{1}{ab}\left(x_{e}+\frac{a}{2}\right)\left(y_{e}+\frac{b}{2}\right), & \phi_{4}^{e} & =-\frac{1}{ab}\left(x_{e}-\frac{a}{2})(y_{e}+\frac{b}{2}\right). \end{array} \end{array}$$

Using the shape functions above, $\phi_{j}$ defines a mapping between the continuous field and four-element vertices through

(10) $$\phi_{j}=\left[0\;\ldots \;\phi_{1}^{e}\;\phi_{2}^{e}\;\phi_{3}^{e}\;\phi_{4}^{e}\;\ldots \;0\right],\;\phi_{j}\in {\left[0,1\right]}^{1\times n}.$$

In this manner, a measurement $y_{j}\left(t_{k}\right)$ can be expressed in terms of the element grid approximation, writing

(11) $$y_{j}\left(q_{j}\right)=\phi_{j}\left(q_{j}\right)f+v,\;\;v∼N\left(0,\sigma_{y}^{2}\right).$$

Moreover, the mapping of *N* measurements at time $t_{k}$ is obtained as $\Phi_{k}={\left[\phi_{1}{\left(t_{k}\right)}^{⊤},\ldots ,\phi_{N}{\left(t_{k}\right)}^{⊤}\right]}^{⊤}$. Further we define the mapping $\Phi_{1:k}={\left[\Phi_{1}^{⊤},\ldots ,\Phi_{k}^{⊤}\right]}^{⊤}$.

### 3.2. Gaussian Markov Random Field Regression

Gaussian Markov random fields define a conditional auto-regressive (CAR) process. A process is a CAR(*j*) process, if the expectation of a process value is fully defined through the next *j* adjacent graph vertices. The Markov assumption enables the direct construction of a sparse precision matrix. Given a labeled graph G=(V,E) with vertices V={1,…,n} and edges E, a probabilistic graphical model η defines a GMRF, if the edges E are chosen such that there is no edge between node *i* and *j*, if $\eta_{i}\;⊥\;\eta_{j}\;|\;\eta_{-ij},$ in which −ij denotes the nodes adjacent to *i* and *j*, respectively [25]. The pairwise conditional independence properties of x on G are inherent in the subdiagonal entries of the precision matrix Λ. We refer the reader to [25] for an in-depth discussion of GMRFs.

**Figure 4:**
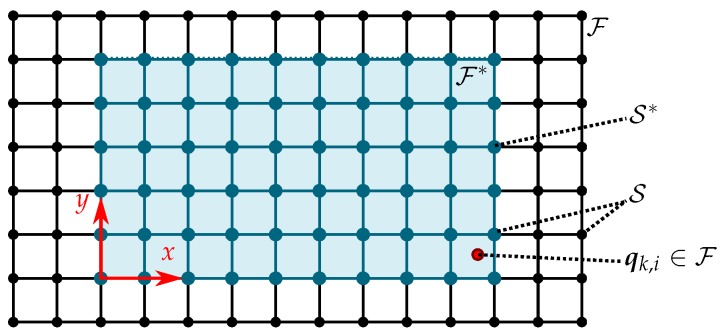
The agent’s position $q_{k,i}$ at the discrete time step *k* lies in a spatial field $F^{*}$ with coordinate values *x* and *y*. The field $F^{*}$ can be extended to F and is then discretized into a regular grid S to enable the construction of a GMRF and to compensate for boundary effects.

In order to construct the GMRF, the continuously indexed spatial field $F^{*}\subset R^{d}$ is discretized into a labeled undirected spatial graph with $n^{*}$ vertex positions $S^{*}=x_{1},\ldots ,x_{n_{*}}$, where $x_{i}$ denotes the i-th field vertex position (Note that in this work the scalars *x* or *y* denote the spatial position coordinates of a two-dimensional spatial position vector x, while a bold y represents an environmental field observation vector.). The set of field locations $S^{*}$ is extended to S with vertex positions $S=x_{1},\ldots ,x_{n}$, as depicted in Figure 4, to compensate boundary effects occurring due to the GMRF approximation. Then on S, a GMRF η is constructed using the SPDE approach proposed by [12].

Let η∼N(0,Σ) be a GP on $R^{2}$ defined by the Matérn covariance function defined as

(12) $$k_{\mathrm{Matérn}}\left(x,x^{\prime }\right)=\sigma_{f}^{2}\;\frac{2^{1-\nu }}{\Gamma (\nu )}{\left(\kappa \;∥x-x^{\prime }∥\right)}^{\nu }\;K_{\nu }\left(\kappa \;∥x-x^{\prime }∥\right),$$

in which ∥·∥ denotes the Euclidean distance in $R^{d}$ and $K_{\nu }$ the modified Bessel function of the second kind. The GMRF $\eta ∼N(0,\Lambda^{-1})$ defined on a regular two-dimensional lattice approximates a Matérn GP for ν=0 if the Gaussian full conditionals read

(13) $$\begin{array}{c} E\left(\eta |\eta_{-ij},\theta \right)=\frac{1}{a}\left(\eta_{i-1,j}+\eta_{i+1,j}+\eta_{i,j-1}+\eta_{i,j+1}\right)=\frac{1}{a}\left(\begin{array}{ccc} \circ & • & \circ \\ • & \circ & •\\ \circ & • & \circ \end{array}\right),\;\mathrm{Pre}\left(\eta |\eta_{-ij},\theta \right)=a\tau . \end{array}$$

**Figure 5:**
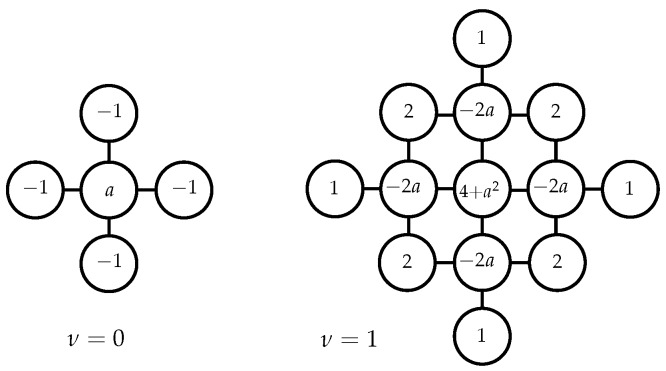
Non-zero elements of a column of the precision matrix Λ associated with one random variable and its neighbor vertices on a regular two-dimensional GMRF lattice.

For the case of ν=1, the Gaussian full conditionals read

(14) $$\begin{array}{c} E\left(\eta |\eta_{-ij},\theta \right)=\frac{1}{4+a^{2}}\left(2a\;\begin{array}{ccccc} \circ & \circ & \circ & \circ & \circ \\ \circ & \circ & • & \circ & \circ \\ \circ & • & \circ & • & \circ \\ \circ & \circ & • & \circ & \circ \\ \circ & \circ & \circ & \circ & \circ \end{array}-2\;\begin{array}{ccccc} \circ & \circ & \circ & \circ & \circ \\ \circ & • & \circ & • & \circ \\ \circ & \circ & \circ & \circ & \circ \\ \circ & • & \circ & • & \circ \\ \circ & \circ & \circ & \circ & \circ \end{array}-1\;\begin{array}{ccccc} \circ & \circ & • & \circ & \circ \\ \circ & \circ & \circ & \circ & \circ \\ • & \circ & \circ & \circ & •\\ \circ & \circ & \circ & \circ & \circ \\ \circ & \circ & • & \circ & \circ \end{array}\right)\begin{array}{c} ,\;\mathrm{Pre}\left(\eta |\eta_{-ij},\theta \right)=\left(4+a^{2}\right)\tau , \end{array} \end{array}$$

with $a=\kappa^{2}+4$ and $\theta ={\left[\tau ,\kappa \right]}^{⊤}\in R_{>0}^{2}$ being hyperparameters of the model. The additional hyperparameter *τ* adjusts the GMRF’s signal variance independent of *κ*. The proof of Equations (13) and (14) for the general case of irregular grids is stated in [12]. Figure 5 illustrates the correspondence between the spatial lattice locations and the values in each column of Λ using the previously presented construction scheme.

When designing the GMRF precision matrix, the full conditionals for the border vertices of the GMRF grid affect the estimation result considerably. Three commonly used boundary conditions are the Dirichlet, Neumann, and torus boundary condition [25].

#### 3.2.1. Sequential GMRF Regression

In this Subsection, the GMRF regression algorithm proposed in [13] is extended to enable spatial process estimation with continuous observations. The values of the field are represented by the latent variable $z_{i}=z\left(s_{i}\right)\in R$. The latent variables are expressed using a global linear model, such that

(15) $$\begin{array}{cc} z_{i} & =\mu \left(s_{i},\beta \right)+\eta_{i}\;\forall \;1\leq i\leq n, \end{array}$$

(16) $$\begin{array}{cc} \mu \left(s_{i},\beta \right) & =m^{⊤}\beta . \end{array}$$

Hereby, $m={\left[m_{1}\left(s_{i}\right),\ldots ,m_{p}\left(s_{i}\right)\right]}^{⊤}\in R^{p}$ denotes the regression function vector and the vector $\beta ={\left[\beta_{1},\ldots ,\beta_{p}\right]}^{⊤}$ contains the unknown regression coefficients. The field belief on the lattice is denoted as $z={\left[z_{1},\ldots ,z_{n}\right]}^{⊤}$. The small-scale correlations of the field are modeled through the zero-mean GMRF $\eta ∼N(0,\Lambda_{\eta |\theta })$. We initialize the GMRF precision matrix $\Lambda_{\eta |\theta }^{-1}$ with the full conditionals as defined in (13) and (14). A zero-mean Gaussian prior is assumed on the regression coefficients $\beta ∼N(0,T^{-1})$ to estimate the regression coefficients as a function of z and θ, where $T^{-1}$ is initialized as a diagonal matrix with large diagonal elements. The probability distribution of the full latent field $\bar{z}={\left[z^{⊤},\beta^{⊤}\right]}^{⊤}\in R^{n+p}$ reads

(17) $$\begin{array}{cc} p(\bar{z},\theta ) & =p(z|\beta ,\theta )\;p(\beta ),\\ & \propto \exp\left(-\frac{1}{2}{\left(z-m\beta \right)}^{⊤}\Lambda_{\eta |\theta }\left(z-m\beta \right)-\frac{1}{2}\beta^{⊤}T\beta \right),\\ & =\exp\left(-\frac{1}{2}{\bar{z}}^{⊤}\Lambda_{\bar{z}|\theta }\bar{z}\right), \end{array}$$

with precision matrix $\Lambda_{\bar{z}|\theta }\in R^{\left(n+p)\times (n+p\right)}$ being defined as

(18) $$\Lambda_{\bar{z}|\theta }=\left[\begin{array}{cc} \Lambda_{\eta |\theta } & -\Lambda_{\eta |\theta }m\\ -m^{⊤}\Lambda_{\eta |\theta } & m^{⊤}\Lambda_{\eta |\theta }m+T \end{array}\right].$$

By exploiting the GMRF’s *canonical form*, only the current available measurements $y_{k}$ are required to sequentially update the conditional precision matrix $\Lambda_{\bar{z}|\theta ,y_{1:k}}≜\Lambda_{k|\theta }$ and the *canonical mean* $b_{k}=\Lambda_{k|\theta }\mu_{k|\theta }$. A sequential updating algorithm is obtained by inserting the canonical mean definition into the formula for conditioning of a multivariate Gaussian distribution, such that

(19) $$\begin{array}{cc} p(\bar{z}|\theta ,y_{1:k}) & =N(\Lambda_{k|\theta }^{-1}b_{k},\Lambda_{k|\theta }^{-1}), \end{array}$$

(20) $$\begin{array}{cc} \Lambda_{k|\theta } & =\Lambda_{\bar{z}|\theta }+\frac{1}{\sigma_{y}^{2}}\Phi_{1:k}^{⊤}\Phi_{1:k}=\Lambda_{k-1|\theta }+\frac{1}{\sigma_{y}^{2}}\Phi_{k}^{⊤}\Phi_{k}, \end{array}$$

(21) $$\begin{array}{cc} b_{k} & =\frac{1}{\sigma_{y}^{2}}\Lambda_{k|\theta }\Lambda_{k|\theta }^{-1}\Phi_{1:k}^{⊤}y_{1:k}=b_{k-1}+\frac{1}{\sigma_{y}^{2}}\Phi_{k}^{⊤}y_{k}, \end{array}$$

with initial conditions $\Lambda_{0|\theta }=\Lambda_{\bar{z}|\theta }$ and $b_{0}=0$. Note that the shape function vectors are extended by a zero vector of length *p* such that $\Phi_{k}\in R^{n+p}$. The final sequential GMRF regression algorithm is summarized in Algorithm 1. In order to obtain the variance vector $\mathrm{diag}(\Lambda_{k|\theta }^{-1})$ of the full latent field, without calculating the inverse of the precision matrix, the Sherman–Morrison formula is used, Line 13. The complete derivation is outlined in Appendix A. For the sake of clarity, the notation for the conditioning of the GMRF matrices on the hyperparameters θ is omitted. It is worth mentioning that adding the product $\Phi_{k}^{⊤}\Phi_{k}$ to $\Lambda_{0|\theta }$ does not significantly increase the density of the initial precision matrix. Thus, the algorithm has a computational complexity of O(1) over time.

**Algorithm 1** Sequential GMRF Regression**Require:** Hyperparameter vector θ, Extended field grid S, Regression function vector m       Measurement variance $\sigma_{y}^{2}$, $b_{0,0}=0$, $\Lambda_{0,0}≜\Lambda_{\bar{z}}$     
1:compute $\mathrm{diag}\left(\Sigma_{0}\right)=\mathrm{diag}\left(\Lambda_{\bar{z}}^{-1}\right)$2:**for**$k\in Z_{>0}$**do**3:    **for** 1≤j≤N **do**4:        get *j*-th agent location $x_{k,j}$ and measurement $y_{k,j}$5:        compute $\Phi_{k,j}\left(x_{k,j},S\right)$6:        $b_{k-1,j}=b_{k-1,j-1}+\frac{1}{\sigma_{y}^{2}}\Phi_{k,j}^{⊤}y_{k}$  7:        $\Lambda_{k-1,j}=\Lambda_{k-1}+\sum_{l=1}^{N}\frac{1}{\sigma_{y}^{2}}\Phi_{k,l}^{⊤}\Phi_{k,l}$  8:        $h_{k,j}=\Lambda_{k-1,j}^{-1}\Phi_{k,j}^{⊤}$9:    **end for**10:    $b_{k,0}=b_{k-1,N}$11:    $\Lambda_{k,0}=\Lambda_{k-1,N}$12:    $\mu_{k}=\Lambda_{k,0}^{-1}b_{k,0}$13:    $\mathrm{diag}\left(\Sigma_{k}\right)=\mathrm{diag}\left(\Sigma_{k-1}\right)-\sum_{l=1}^{N}\frac{h_{k,l}\circ h_{k,l}}{\sigma_{y}^{2}+\Phi_{k,l}h_{k,l}}$14:**end for**


#### 3.2.2. Hyperparameter Estimation for Sequential GMRF Regression

A possible straightforward extension of the proposed model, is described in Xu et al. [13]. In this work, hyperparameter estimation is incorporated by defining the maximum a posteriori distribution p(θ|y)∼p(y|θ)p(θ) with p(θ) being a uniform prior distribution over a discrete set of hyperparameter combinations. Approximating the integral by a discrete sum decreases the computational load compared to a numerical integration over p(θ|y). Furthermore, such an approach scales linearly with the number of hyperparameter combinations and can be extended to incorporation of continuous measurements. However, the method requires that the set of hyperparameters are chosen a priori.

### 3.3. Kalman Regression for Field Estimation

**Figure 6:**
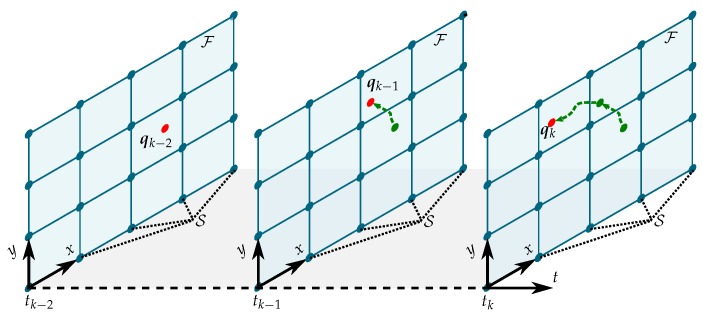
An agent takes measurements at the position q while maneuvering through a field F. The environmental field is discretized into a regular lattice with the set of locations S. The STKF random variables $\hat{f}\left(t\right)$ estimate the spatio-temporal process f(t) on S.

The Kalman regression model by [15], also referred to as spacetime Kalman filtering (STKF), incorporates off-grid measurements by adding new grid vertices to the belief lattice. In the following, we propose the concept of weighted shape functions to fuse new observations from continuous space into the already existing neighboring vertices of the discrete grid, see Figure 6. This allows us to keep the number of vertices and their spatial density constant. In order to make this article self-sufficient, we briefly summarize the derivation of the STKF.

#### 3.3.1. Process Model

Given the spatio-temporal physical process f(x,t), its covariance function K(·) is assumed to be separable in time and space, as well as stationary in time $K\left(x,x^{\prime },t,t^{\prime }\right)=K_{s}\left(x,x^{\prime }\right)K_{t}\left(t,t^{\prime }\right)$. Therefore, the power spectral density (PSD) $S_{r}\left(\omega \right)=W\left(i\omega \right)W\left(-i\omega \right)$ of the temporal covariance $K_{t}$ can be approximated by a rational function of order 2r. As rational functions are universal function approximators, arbitrary (non-stationary) temporal spectra can be approximated by increasing *r*.

The individual temporal process component $z_{i}\left(t\right)$ defined by $K_{t}$ is represented by a continuous state space model $S_{i}$ in companion form using the Wiener–Khinchin theorem and realization theory, such that

(22) $$S_{i}:\left\{\begin{array}{c} {\dot{s}}_{i}\left(t\right)=Fs_{i}\left(t\right)+Gw_{i}\left(t\right),\\ z_{i}\left(t\right)=Hs_{i}\left(t\right), \end{array}\right.\;i\in \left\{1,\ldots ,n\right\}.$$

where w(t)∼N(0,I) and the matrices F, G, and H are in companion form

(23) $$\begin{array}{c} F=\left[\begin{array}{ccccc} 0 & 1 & 0 & \cdots & 0\\ 0 & 0 & 1 & \cdots & 0\\ & & \ddots & & \\ 0 & 0 & 0 & \cdots & 1\\ -a_{0} & -a_{1} & -a_{2} & \cdots & -a_{r-1} \end{array}\right],\;G=\left[\begin{array}{c} 0\\ 0\\ \vdots \\ 0\\ 1 \end{array}\right],\;H=\left[\begin{array}{ccccc} b_{0} & b_{1} & b_{2} & \cdots & b_{r-1} \end{array}\right]. \end{array}$$

The initial state yields $s\left(0\right)∼N\left(0,\Sigma_{0}\right)$. The covariance matrix $\Sigma_{0}$ is obtained as the solution to the Lyapunov equation $F\Sigma_{0}+\Sigma_{0}F^{⊤}+GG^{⊤}=0$. Note that s(t) does not provide a directly intuitive physical interpretation. The temporal process component is obtained as $z\left(t\right)={\left[z_{1}\left(t\right),\ldots ,z_{n}\left(t\right)\right]}^{⊤}\in R^{n}$. The spatial covariance matrix ${\bar{K}}_{S}$ is computed on S through the spatial covariance function $K_{s}$. Finally, the process on S is obtained by spatially correlating all $z_{i}\left(t\right)$ through the Cholesky factorization ${\bar{K}}_{S}^{1/2}$ of ${\bar{K}}_{S}$, reading

(24) $$f\left(t\right)={\bar{K}}_{S}^{1/2}z\left(t\right).$$

**Figure 7:**
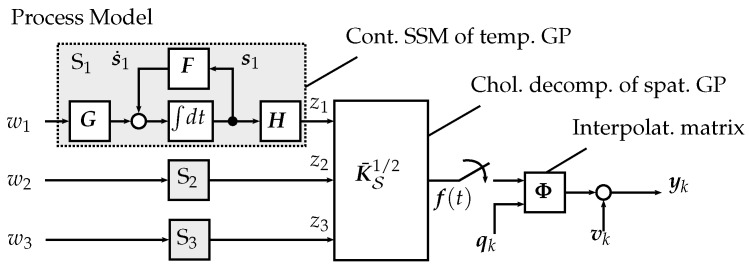
Block diagram of the process model on a field lattice with three vertices.

The spatio-temporal process model is depicted in Figure 7. With $s\left(t\right)={\left[s_{1}^{⊤}\left(t\right),\ldots ,s_{n}^{⊤}\left(t\right)\right]}^{⊤}\left(t\right)\in R^{n\times r}$ and process noise $w\left(t\right)=\left[w_{1}\left(t\right),\ldots ,w_{n}\left(t\right)\right]\in R^{n}$, Equation (22) is condensed to

(25) $$S:\left\{\begin{array}{c} \dot{s}\left(t\right)=\left(I⊗F\right)s\left(t\right)+\left(I⊗G\right)w\left(t\right),\\ f\left(t\right)={\bar{K}}_{S}^{1/2}\left(I⊗H\right)s\left(t\right), \end{array}\right.$$

in which the Kronecker product is denoted by ⊗. The previous equations are discretized to enable numerical implementation. Thereby, the discrete time step $t_{k}$ is abbreviated as *k* with a single time step being $T_{k}=t_{k}-t_{k-1}$. The discrete process model of (25) reads

(26) $$\left\{\begin{array}{c} s_{k+1}=As_{k}+w_{k},\\ y_{k}=C_{k}s_{k}+v_{k}. \end{array}\right.$$

The discrete state transition matrix is obtained as

(27) $$A=\exp\left(I⊗F\right)T_{k}\in R^{rn\times rn}.$$

The zero mean Gaussian noise $w_{k}\in R^{rn}$ is defined by the covariance matrix $Q_{k}=I⊗{\bar{Q}}_{k}$ with

(28) $${\bar{Q}}_{k}=\int_{0}^{T_{k}}\left(\exp\left(F\tau \right)GG^{⊤}{(\exp\left(F\tau \right)}^{⊤}\right)d\tau .$$

The measurement noise vector $v_{k}={\left[v_{k,1},\ldots ,v_{k,N}\right]}^{⊤}$ is defined as $v_{k}∼N\left(0,\Sigma_{y}\right)$ with $\Sigma_{y}=\sigma_{y}^{2}I\in R^{N\times N}$.

The discrete observation matrix is obtained as

(29) $$C_{S,k}={\bar{K}}_{S}^{1/2}\left(I⊗H\right)\in R^{N\times rn}.$$

In contrast to [15], where new vertices are initialized to include off-grid information, we map the observation $y_{k}$ collected at timestep *k* at position $q_{k}$ to neighboring belief vertices through (11). Using the measurement interpolation matrix $\Phi_{k}$, we obtain a transformation from the discrete belief lattice to a continuous field measurement as

(30) $$C_{k}=\Phi_{k}{\bar{K}}_{S}^{1/2}\left(I⊗H\right)=\Phi_{k}C_{S,k}\in R^{N\times rn},\mathrm{with}\;\Phi ={\left(\Phi_{1}^{⊤},\ldots ,\Phi_{N}^{⊤}\right)}^{⊤}.$$

#### 3.3.2. Kalman Regression

As the temporal process model in (26) is known and linear, a Kalman filter scheme can be used to estimate the evolution of the temporal process component $s_{k}$ by incorporating observation $y_{k+1}$ [26]. Furthermore, if the noise is assumed to be zero mean and Gaussian distributed, the Kalman filter estimates are optimal in a mean-square sense. The STKF belief model is summarized in Algorithm 2.

Given the state space model in (26), the temporal state component at time step k+1 evolves in time according to $s_{k+1}∼N\left(A_{k}s_{k},Q_{k}\right)$, where $Q_{k}$ is the corresponding process noise matrix. The measurement obtained from the real process is assumed to result from an affine transformation of the temporal state component, reading $y_{k+1}∼N\left(C_{k+1}s_{k+1},\Sigma_{y}\right).$

In a first step, the Kalman filter *predicts* the temporal process component at the next time step ${\hat{s}}_{k+1|k}$ based on the previous estimated temporal state component ${\hat{s}}_{k|k}$, such that $s_{k+1|k}∼N\left({\hat{s}}_{k+1|k},\Sigma_{k+1|k}\right).$ The state mean prediction ${\hat{s}}_{k+1|k}$ and predicted state covariance matrix $\Sigma_{k+1|k}$ are depicted in lines 9 and 10 of Algorithm 2 respectively.

In the second step, the Kalman filter *updates* the temporal state component ${\hat{s}}_{k+1|k+1}$ by conditioning the RV on $y_{k+1}$, such that $s_{k+1|k+1}∼N\left({\hat{s}}_{k+1|k+1},\Sigma_{k+1|k+1}\right).$ The equations for the updated state and covariance matrix are stated in lines 11 to 13 of Algorithm 2.

**Figure 8:**
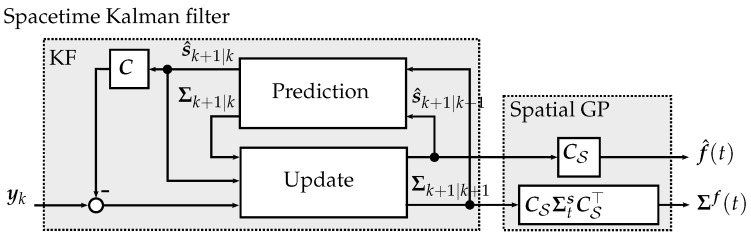
Block diagram of the spacetime Kalman filter.

Figure 8 illustrates the STKF model. The process model consists of the state space models of the temporal process components $z_{i}\left(t\right)$, which are correlated using the product of the Cholesky decomposition of the spatial covariance matrix $C_{S}={\bar{K}}_{S}^{1/2}\left(I⊗H\right)$. The Kalman filter predicts the next temporal process component, which is then updated using new measurements $y_{k}$.

**Algorithm 2** Kalman regression**Require:**(F,G,H) state-space model of $S_{r}\left(\omega \right)$, measurement noise variance $\sigma_{y}^{2}$, input location set S, spatial, time kernels $K_{s}\left(\cdot ,\cdot \right)$, and h(·)   
1:$\hat{s}\left(0|0\right)=0$ and $\Sigma \left(0|0\right)=I⊗\Sigma_{0}$.2:Compute $\Sigma_{0}$ as solution of $F\Sigma_{0}+\Sigma_{0}F+GG^{⊤}=0$3:Compute $A_{k}$, $Q_{k}$, $\Sigma_{y,k}$ and $C_{S}={\bar{K}}_{S}^{1/2}\left(I⊗H\right)$   4:**for**$t\in R_{>0}$**do**5:    **if** $t\in \;]t_{k},t_{k+1}[$ **then** {open-loop prediction}6:        $\hat{s}\left(t\right)=\left(\exp^{(I⊗F)\tau }\right)\Sigma_{k|k}{\left(\exp^{(I⊗F)\tau }\right)}^{⊤}$7:    **else if** $t=t_{k+1}$ **then** {Kalman estimation}8:        Compute $\Phi (q_{k+1})$ and $C_{k+1}=\Phi \left(q_{k+1}\right)C_{S}$        - Prediction step:9:          ${\hat{s}}_{k+1|k}=A_{k}{\hat{s}}_{k|k}$10:          $\Sigma_{k+1|k}=A_{k}\Sigma_{k|k}A_{k}^{⊤}+Q_{k}$        - Update step:11:          $L_{k+1}=\Sigma_{k+1|k}C_{k+1}^{⊤}{\left(C_{k+1}\Sigma_{k+1|k}C_{k+1}^{⊤}+\Sigma_{y,k+1}\right)}^{-1}$12:          ${\hat{s}}_{k+1|k+1}={\hat{s}}_{k+1|k}+L_{k+1}\left(y_{k+1}-C_{k+1}{\hat{s}}_{k+1|k}\right)$13:          $\Sigma_{k+1|k+1}=\left(I-L_{k+1}C_{k+1}\right)\Sigma_{k+1|k}$14:        $\hat{s}\left(t\right)={\hat{s}}_{k+1|k+1}$15:        $\Sigma^{s}\left(t\right)=\Sigma_{k+1|k+1}$16:    **end if**     - Process estimate:17:      $\hat{f}\left(t\right)=C_{S}\hat{s}\left(t\right)$18:      $\Sigma^{f}\left(t\right)=C_{S}\Sigma^{s}\left(t\right)C_{S}^{⊤}$19:**end for**


The computational complexity of Algorithm 2 is dominated by the inverse computation of the Kalman gain in line 12. The computational complexity of the STKF algorithm is bounded by

(31) $$O(r\cdot n\cdot N+N^{3}+n\cdot P),$$

in which *r* is the order of a single state space model in (23), *n* is the number of vertices of S, while *P* is the number of open-loop predictions performed, and *N* is the number of agents collecting measurements at each discrete time step [15]. In this work, we do not perform any open-loop predictions, thus *P* is zero. Note that all variables in (31) are assumed to be constant over time. If spatial hyperparameter estimation is performed, the Cholesky transformation of the new spatial covariance matrix needs to be computed. In this case, the computational load is dominated by the computation of the spatial Cholesky decomposition being $O(n^{3})$.

#### 3.3.3. Hyperparameter Estimation in Kalman Regression

In the STKF, the spatial Kernel hyperparameters can be estimated using a standard estimation method for GPs, such as maximum a posteriori estimation. However, GP hyperparameter estimation methods have the disadvantage of suffering from the big-*n* problem. In this sense, finding an optimization method that enables spatial hyperparameter estimation represents a challenge yet to be solved. As a state space model approximates the temporal process component, the temporal process hyperparameters can be estimated using methods developed for parameter estimation in finite state space models, as pointed out in [27]. Such methods inherit the advantage of having linear time computational complexity.

## 4. Path Integral Control for Exploratory Path Planning

The final path planning algorithm is summarized in Algorithm 3. In a discrete receding horizon formulation, the optimal control vector is recomputed at each sampling instance, while only the first control input is applied to the path planning model to generate the next way-point.

In the first initialization of the algorithm the initial control sequence $u_{0:(H-1)}\left(k=0\right)$ is assumed to be the null vector. Afterwards, at each subsequent planning step, the initial control sequence is set to $u_{0:(H-1)}\left(k\right)={\left[u_{1:(H-2)}{\left(k-1\right)}^{⊤},0\right]}^{⊤}$. With $u_{0:(H-1)}\left(k\right)$ and the sampled exploration noise $\varepsilon_{0:(H-1),\ell }$ the *ℓ*-th path roll-out is computed in line 6 of Algorithm 3.

Exploration of underwater environmental fields is often conducted by underwater robots whose propulsion system produces mainly forward-directed thrust, which allows higher cruising speeds and, thus, faster exploration. These robots typically come with non-holonomic dynamics, e.g., the HipppoCampus micro underwater robot [3]. Hence, analog to [23], we use an unicycle model to make use of the dynamic constraints and efficiently generate path roll-outs. The model reads

(32) $$\begin{array}{cc} x_{k+1} & =\left[\begin{array}{c} x_{k+1}\\ y_{k+1}\\ \alpha_{k+1} \end{array}\right]=\left[\begin{array}{c} v\cos(\alpha_{k})\\ v\sin(\alpha_{k})\\ f^{\left(c\right)} \end{array}\right]\Delta t+\left[\begin{array}{c} 0\\ 0\\ g^{(c)⊤} \end{array}\right]\left(u_{k}\Delta t+\varepsilon_{k}\sqrt{\Delta t}\right), \end{array}$$

with the directly controllable system dynamics $f^{c}{}_{\mathrm{robot}}=0$, as well as the control transition matrix $g^{(c)⊤}=1$ being deterministic. Since $g^{(c)⊤}$ is scalar, the weighted control projection matrix also reduces to a scalar $M_{t_{j}}=1$.

The cost for the *ℓ*-th path segment $\tau_{i:H,\ell }$ is computed in line 7 of Algorithm 3. Note that if we average over $M_{t_{i}}u_{0:(H-1)}$ the algorithm could become unstable. As [21] points out, the matrix $M_{t_{i}}$ is a projection of $u_{0:(H-1)}$ onto the column space of $g_{t_{j}}$ weighted by the metric $R^{-1}$. A multiplication with $M_{t_{i}}$ results in a decreasing $u_{0:(H-1)}$. The state cost $q_{i,\ell }$ is set to be the predictive variance of the belief model at the associated state.

**Figure 9:**
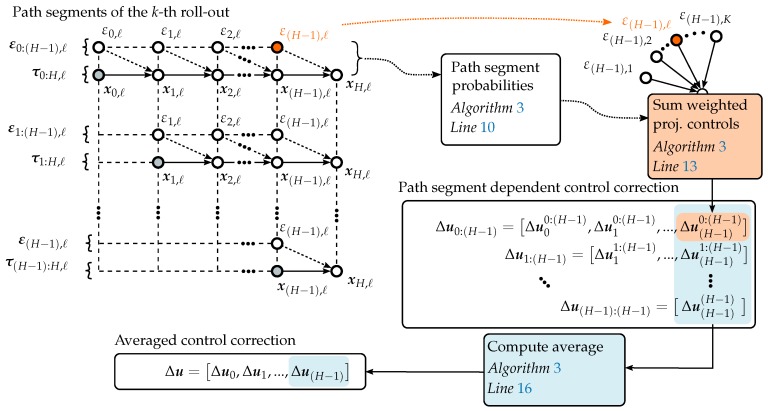
Schematic visualization of the computational steps involved in computing the averaged control correction from *K* sampled path roll-outs of length *H* using the PI${}^{2}$.

Afterwards, the probability of each path segment $P(\tau_{i:H,\ell })$ is obtained by normalizing each probability measure of $\tilde{S}\left(\tau_{i:H,\ell }\right)$ through the sum of path segment probabilities of all roll-outs in line 10. In this line, λ is a sensitivity parameter that is eliminated by subtracting a constant term from $\tilde{S}\left(\tau_{i:H,\ell }\right)$, writing

(33) $$\exp\left(-\frac{1}{\lambda }\tilde{S}\left(\tau_{i:H,\ell }\right)\right)=\exp\left(-c\frac{\tilde{S}\left(\tau_{i:H,\ell }\right)-\min\tilde{S}\left(\tau_{i:H,\ell }\right)}{\max\tilde{S}\left(\tau_{i:H,\ell }\right)-\min\tilde{S}\left(\tau_{i:H,\ell }\right)}\right),$$

with c=10, as proposed by [21]. Figure 9 illustrates the computation of the path segment dependent control correction through line 13 and subsequently the computation of the averaged control vector through line 16. For all path segments of equal length, the exploration noise at each step of the path segment of equal length are weighted by $P(\tau_{i:H,\ell })$ and summed up. This results in the weighted path segment dependent control correction, line 13 of Algorithm 3. In this equation, the lower index defines the relative position in the path segment, while the higher index defines to which path segment the vector entry belongs. Per definition, the index number of the last sample path location is equal to $H=t_{H}/\Delta t$. It follows that $\Delta u_{0:(H-1)}$ is of size H×1 and $\Delta u_{i:(H-1)}$ is of size (H−i)×1. Hence, line 13 computes an optimal control correction vector $\Delta u_{i:(H-1)}$ for each path segment over all *K* roll-outs.

**Algorithm 3** PI${}^{2}$ for path planning**Require:** Cost function $r_{k}=q_{k}+u^{⊤}Ru$, unicycle exploration policy $x_{k+1}\left(u_{k},x_{k},\Delta t\right)$, exploration noise variance $\Sigma_{\varepsilon }$, sampling time Δt, initial optimal control sequence $u_{0:(H-1)}\left(k=0\right)$, number of sampled paths *K*, control horizon steps *H*, control computation iterations $n_{\mathrm{updated}}$
1:$u_{0:(H-1)}^{\left(\mathrm{start}\right)}\left(k\right)={\left[u_{1:(H-2)}{\left(k-1\right)}^{⊤},0\right]}^{⊤}$  2:$M=\frac{R^{-1}gg^{⊤}}{g^{⊤}R^{-1}g}$ (Weighted control projection matrix)3:**for**$1\leq \left(...\right)\leq n_{\mathrm{updated}}$**do**4:    **for** 1≤ℓ≤K **do**     5:        - Sample exploration noise: $\varepsilon_{0:(H-1),\ell }∼N\left(0,\Sigma_{\varepsilon }\right)$6:        - Compute path roll-outs: $\tau_{0:H,\ell }\left(x_{t_{j}},u_{0:(H-1)},\varepsilon_{0:(H-1),\ell },\Delta t\right)$7:        - Compute cost of paths segments:            $\tilde{S}\left(\tau_{i:H,\ell }\right)=\sum_{h=i}^{H}q_{h,\ell }+\frac{1}{2}\sum_{h=i-1}^{H-1}\frac{1}{2}{\left(u_{h}+M_{h,\ell }\varepsilon_{h,\ell }\right)}^{⊤}R\left(u_{h}+M_{h,\ell }\varepsilon_{h,\ell }\right)$8:    **end for**9:    **for** 1≤ℓ≤K **do**10:        - Path segment probabilities: $P\left(\tau_{i:H,\ell }\right)=\frac{\exp(-\frac{1}{\lambda }\tilde{S}\left(\tau_{i:H,\ell }\right))}{\sum_{k=1}^{K}\exp\left(-\frac{1}{\lambda }\tilde{S}\left(\tau_{i:H,\ell }\right)\right)}$11:    **end for**12:    **for** 0≤i≤(H−1) **do**13:        - Path segment dependent control correction: $\Delta u_{i:(H-1)}=\sum_{\ell =1}^{K}P\left(\tau_{i:H,\ell }\right)M\varepsilon_{i:(H-1),\ell }$14:    **end for**15:    **for** 0≤i≤(H−1) **do**16:        - Averaged control correction: ${\left[\Delta u\right]}_{i}=\frac{\sum_{h=0}^{H-1}\left(\left(H-h\right){\left[\Delta u_{h:(H-1)}\right]}_{i}\right)}{\sum_{h=0}^{H-1}\left(H-h\right)}$17:    **end for**18:    $u_{0:(H-1)}^{\left(\mathrm{new}\right)}=u_{0:(H-1)}+\Delta u$19:**end for**


Subsequently, the path segment dependent control vectors $\Delta u_{i:(H-1)}$ are weighted by the trajectory length and averaged in line 16. In this manner, for the time step (H−1), *K* potential control corrections are computed and averaged to obtain a single control correction. The obtained control correction Δu is added to the previous control sequence resulting in the new control vector, line 18. The algorithm is repeated until the number of iterations reaches $n_{\mathrm{updates}}$.

## 5. Field Belief Comparison

In this Section, the sequential GMRF regression model stated in Algorithm 1 and the STKF regression model stated in Algorithm 2 are compared for their computational performance time and prediction capabilities. Throughout the analysis, we use the empirical GMRF algorithm proposed by [23] as performance baseline.

### 5.1. Computational Complexity

In the following we analyze the computational complexity of our proposed field belief algorithms. The upper bounds of the belief algorithm’s computational complexity are summarized in Table 1. Thereby, the original empirical GMRF algorithm as proposed in [23] already shows a drastic improvement with regard to processor and memory requirements compared to vanilla GP regression. Nonetheless, the empirical GMRF algorithm still suffers from a linearly increasing computational cost over the number of time steps *k*. The sequential GMRF regression algorithm utilizes the canonical form of the GP, which in combination with a predefined GMRF precision matrix enables a sequential update of the belief. Therefore, the sequential Bayesian GMRF algorithm has a constant computational time with upper bound $O(Nn^{3/2})$ for the two dimensional scenario. In general, the computational time of the GMRF increases with the number of dimensions as the bandwidth of the precision matrix increases [25]. The STKF’s upper bound on the computational complexity is $O(rnN+N^{3})$. For the case of spatial hyperparameter estimation, the STKF’s computational complexity is limited by the computation burden of the spatial Cholesky factorization being $O(n^{3})$ for dense matrices.

### 5.2. Environmental Field Estimation

**Figure 10:**
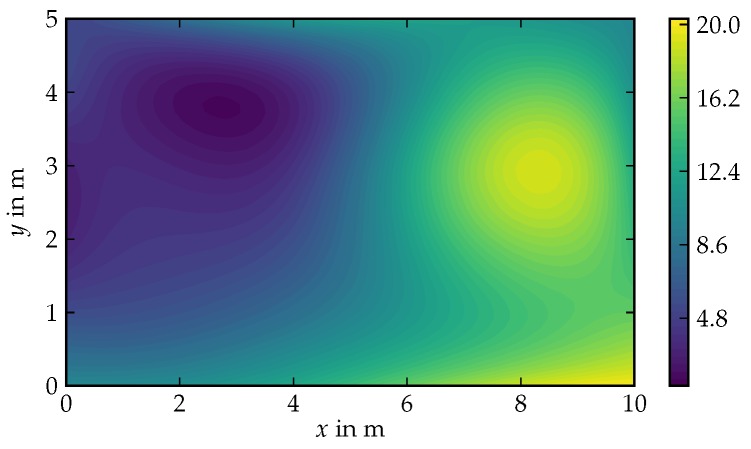
The concentration field used for generating measurements.

The hyperparameter configurations of the individual algorithms and the corresponding acronyms are listed in Table 2. The hyperparameters of the belief model in Table 2 are tuned by hand in order to optimize the approximation result. Hereby, the size of the field lattices are tailored such that the computation time has the same value for all algorithms. Note that GMRF-2 and GMRF-3 use the same regression algorithm, but the used CAR process type, boundary condition, and lattice size differ. During the simulation, measurements are collected from the spatial field depicted in Figure 10. On each belief update step, the next measurement location is chosen to be the point with the highest predictive variance plus a Gaussian noise term with a variance of $0.5\;m^{2}$.

**Figure 11:**
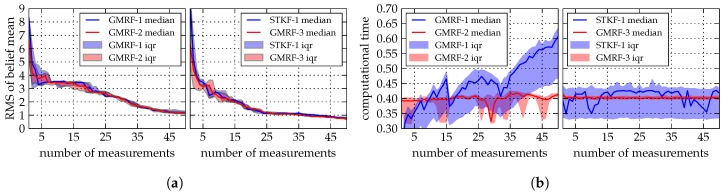
Belief model comparison. (**a**) Median and inter-quarter ranges of the root mean square of the belief algorithms’ predictive variance sum. (**b**) Medians and inter-quarter ranges of the belief algorithms’ computational time over fifty simulation runs.

In order to provide meaningful results the simulation setup is run over 50 individual cycles for each belief algorithm. Figure 11a shows the root mean square (RMS) of the predictive variance sum. We choose the predictive variance sum as belief convergence criteria as it—unlike the empirical mean—is more sensitive to outliers. The convergence behavior of the predictive variance sum’s RMS is utilized as a measure for the exploration performance.

**Figure A1:**
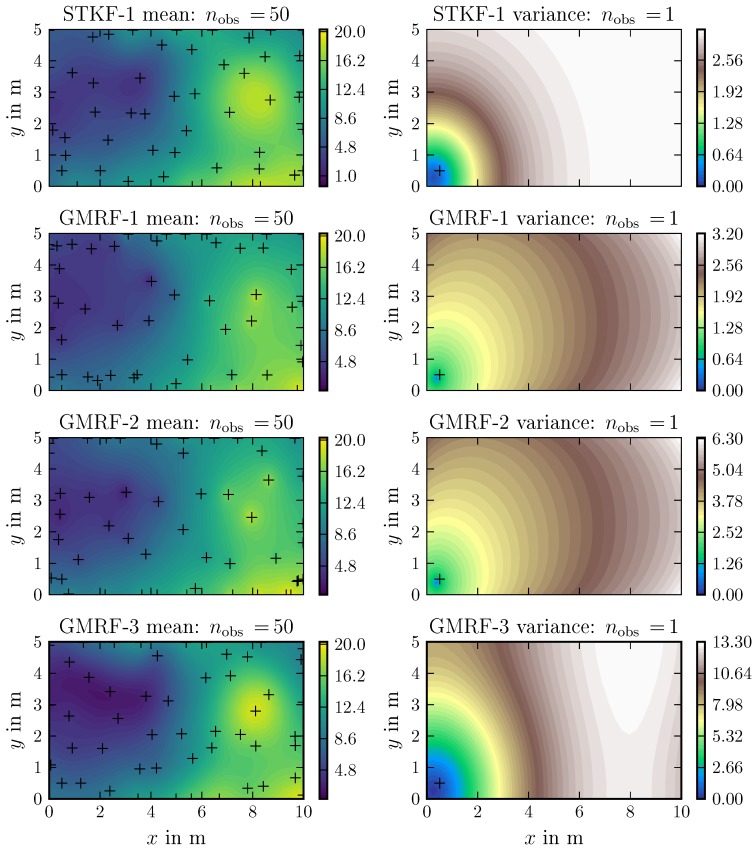
Estimation results of the Gaussian belief models. The parameters of the regression algorithms are denoted in Table 2. **Left:** Predicted field mean of different regression models after fifty observations. In each prediction step the next observation was chosen to be close to the maximum predicted variance. **Right:** Predicted field variance of different belief models after one observation.

Figure A1 in Appendix B illustrates the mean and variance prediction results for the different regression models. Due to the steep correlation structure between known and unknown field values induced by a CAR(1) model, comparatively many measurements are taken in the vicinity of the field boundary. If the padding around the true field GMRF grid is chosen relatively small, distortion effects of the boundary conditions affect the estimation result. The predictive variance of GMRF-1 and GMRF-2 after one measurement shows a non-circular shape, which is induced by the Neumann boundary condition, while the predictive variance of the GMRF-3 after one measurement increases on the edges of the field due to the Torus boundary condition. While designing a GMRF, the dependencies between the field approximation, the used GMRF model, and the chosen boundary condition must be taken into account.

Figure 11b illustrates the median and inter-quarter range (IQR) of the computational time of the different belief models after fifty simulation runs. As expected, the computational time of GMRF-1 increases practically linearly over time due to the increasing number of observations. GMRF-3 has approximately 4500 lattice vertices, while GMRF-2 has approximately 2400 lattice vertices. While GMRF-2 and GMRF-3 both utilize the same regression algorithm with different lattice sizes the computational time almost equals. The same computation time can be attributed to the CAR(2) model used for GMRF-3, which results in a less sparse precision matrix than compared to the CAR(1) model of GMRF-2.

## 6. Analysis of the Exploration Algorithm

In this Section, the STKF belief model is combined with the stochastic controller and the performance of the resulting exploration algorithm, abbreviated as PI-STKF, is analyzed. First, the PI-STKF algorithm is simulated for the scenario of a spatially stationary field as introduced in Section 5.2. Afterwards, the PI-STKF algorithm is simulated in a spatio-temporal exploration scenario, demonstrating the suitability of the developed exploration algorithm for long-term field monitoring tasks. The PI-STKF simulation parameters are listed in Table 3. In order to analyze the effect of different control horizons we consider three robot agents which we abbreviate as ‘Agent-4’, ‘Agent-9’, and ‘Agent-14’, corresponding to their control horizons of $t_{H}=4\;s$, $t_{H}=9\;s$, and $t_{H}=14\;s$ respectively.

In order to analyze the performance of the algorithms completely isolated from external non-reproducible influences, simulations are conducted on a 2.4 GHz Dualcore-CPU ’i5-2430M’ and 8 GB RAM computer where the algorithms are implemented in Python. The computer executes the exploration algorithm at a frequency of approximately 1.5–3 Hz and, thus, sufficiently fast to provide underlying low-level control schemes with the required data. Moreover, it is worth mentioning that the current Python-implementation is not yet speed-optimized such that a sufficiently fast execution time is expected on the micro robot’s onboard hardware.

### 6.1. Analytical Field Exploration

**Figure 12:**
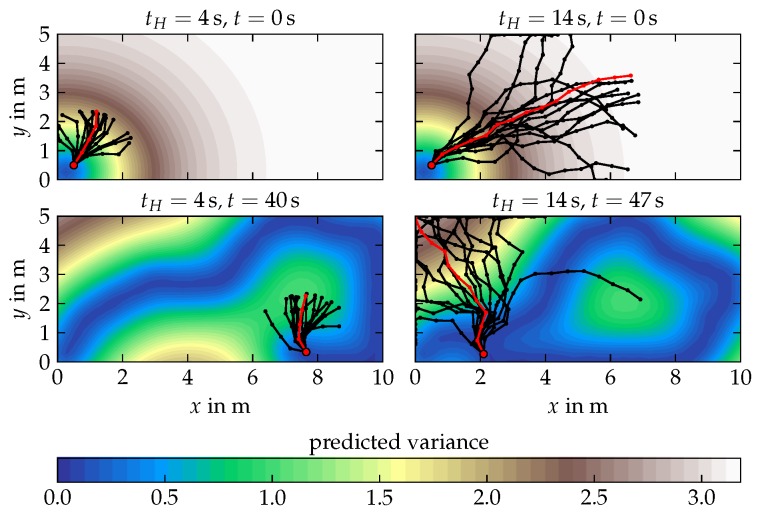
Illustration of the PI-STKF algorithm using different time horizon lengths $t_{H}$. The optimal path (|) starting at the current agent position (•) is computed by an iterative sampling of several potential paths (|) which are then weighted according to their probabilities which depend on the predicted variance.

At every discrete time step, the agent receives a new measurement at its current location from the underlying simulated real field which is depicted in Figure 9. The measurement is perturbed by Gaussian measurement noise with variance $\sigma_{y}^{2}$. The measurement is fed to the STKF belief model. The temporal length scale of the STKF belief model is set to $10^{7}$, such that the conditional variance of the belief model does not noticeably increase over time. The PI${}^{2}$ algorithm utilizes the belief’s conditional (predicted) variance as state cost. Figure 12 illustrates the roll-out sampling step for a control horizon length of $t_{H}=4\;s$ (left) as well as $t_{H}=14\;s$ (right). At t=40s the Agent-4 plans a optimal trajectory towards a local variance maximum. In contrast, Agent-14 takes a path towards the global variance maximum.

**Figure 13:**
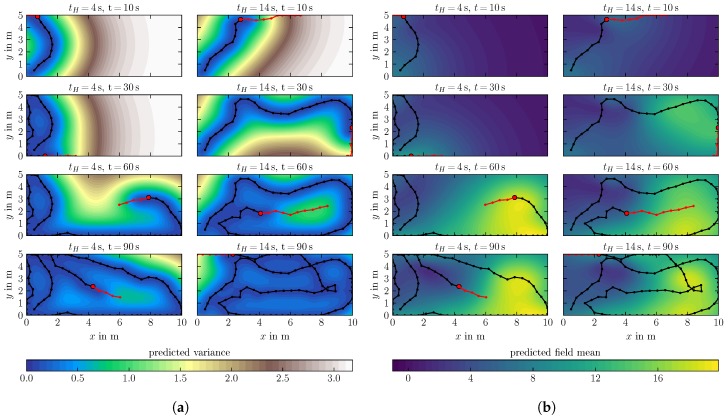
STKF algorithm exploration results for two time horizon lengths $t_{H}=4\;s$ and $t_{H}=14\;s$ and temporal length scale of $10^{7}$ which results in a belief remaining almost constant with respect to time. The agent (•) moves along its trajectory (|) following an optimal path (|) by applying the first optimal control step. The optimal path is computed by PI${}^{2}$ using the conditional variance of the STKF. (**a**) Predicted STKF field variance estimates. (**b**) Predicted STKF field mean estimates.

Figure 13a,b illustrates the exploration result of the PI-STKF algorithm for a short a control horizon of 4 s and a longer horizon of 14 s. As depicted in Figure 12, Agent-4 samples trajectories which are not long enough to reach to the global maximum of the predicted variance field. As a result, Agent-4 follows a rather sub-optimal trajectory from $t_{H}=9\;s$ until t=30s. Furthermore, between t=60s and 90s Agent-4 moves again into the upper left corner, as the rather short sample roll-outs do not provide sufficient information regarding the location of the unknown field values. In contrast, Agent-14 shows a more exploratory behavior. On an intuitive level, one might wonder why Agent-14 first navigates to the upper field boundary, instead of directly maneuvering to the upper right corner of the field. However, even though the prediction horizon is longer than Agent-4’s horizon, it is still possible that the relatively small number of sampled control roll-outs pushes the optimal path towards a temporary less informative path. When Agent-14 almost reaches the upper field boundary, symmetry breaking occurs. Symmetry breaking is a common phenomenon in stochastic optimal control that describes the sudden fixation of the controller towards one sample direction [22]. As the controller’s state cost is the predicted field variance, the agents prioritize a path along the boundary of the field. After t=90s both agents obtained a good belief of the original field process. The effective computational time at each time instance sums up to 0.2 s when using a control horizon of $t_{H}=4\;s$ and 0.3 s and 0.4 s for the control horizons of $t_{H}=9\;s$ and $t_{H}=14\;s$ respectively. Hereby, it is worth mentioning that the controller is implemented in Python using mainly a non-optimized for-loop structure.

**Figure 14:**
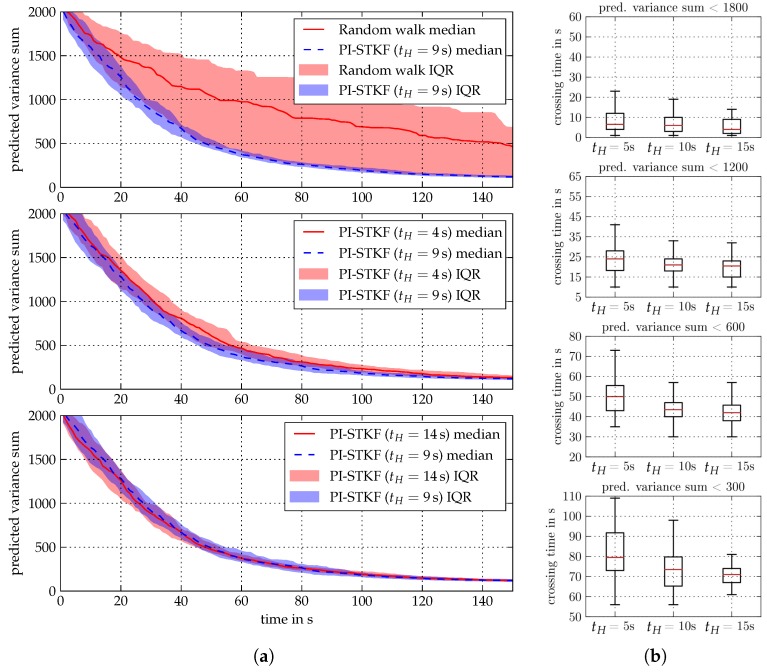
(**a**) Median and IQR of the conditional variance sum of the PI-STKF and a random walk exploration strategy after 50 simulation runs. (**b**) Box plot of the crossing times of a particular conditional variance sum for different control horizons $t_{H}$ over 50 simulation runs.

In order to measure the expected average exploration performance of the PI-STKF algorithm, the simulation with the stochastic field in Figure 10 is repeated 50 times for three different control horizons ($t_{H}=$ 4 s, 9 s, 14 s) where each simulation episode has a length of 150 s. The agent initial position is picked uniformly random within the range of x=0.5m to x=9.5m for the *x*-coordinate while the *y*-coordinate is set to y=0.5m, and the robot’s initial orientation is α=π/2. The obtained results are compared to a random walk exploration strategy as a baseline. Figure 14a illustrates the median and inter-quarter range of the sum of the agent’s conditional variance using STKF-1 as a belief model. Hereby, the stochastic controller consistently outperforms the random walk baseline strategy. In the first 15 s of each simulation the controllers with horizon $t_{H}=4\;s$ and $t_{H}=9\;s$ drive the agent towards the upper field boundary which results in a similar exploration performance. At approximately t=15s the exploration performance of Agent-4 decreases in comparison to the agents with longer control horizons. The information gain per time step in an unexplored field is almost independent from the control horizon when pursuing the the first time steps. However, agents with longer control horizons begin to profit from their far-sight controller as the field exploration mission continues. As a result, when compared to agents with longer horizon Agent-4 tends to maneuver itself on a less informative trajectory. In Figure 14a, the difference in exploration performance between the controller with $t_{H}=9\;s$ and $t_{H}=14\;s$ is small. Nonetheless, exploration missions in larger fields are likely to profit from longer control horizons.

In order to analyze the agent’s exploration efficiency we compare the time spans the agents require reach predefined exploration levels which we represent by the summed predictive variance. Hereby, a crossing time is defined as the time it takes for the agent to drive its field belief predictive variance sum beneath a predefined value. The corresponding crossing times are illustrated in Figure 14b. It can be seen that Agent-9 ($t_{H}=9\;s$) and Agent-14 ($t_{H}=14\;s$) outperform the controller of Agent-4 with a horizon of $t_{H}=4\;s$. Thereby, Agent-9 and Agent-4 show a similar exploration performance down to a predictive variance sum of 600. For lower predictive variances, the controller with $t_{H}=14\;s$ provides a better and more predictable exploration performance. Note that although the difference of the crossing times lies within a range of 20 to 30 s and, thus, comparatively small the effect on the exploration performance will scale with the size of the field and the number of agents.

### 6.2. Spatio-Temporal Field Exploration

**Figure 15:**
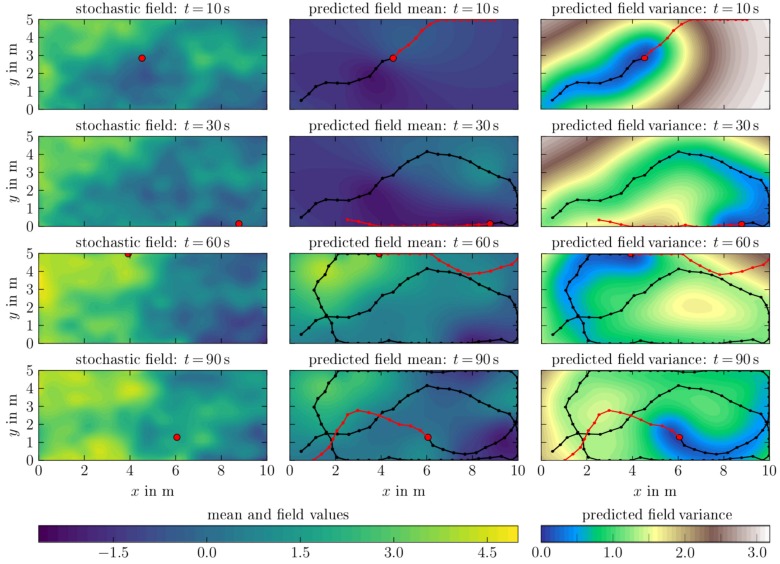
Agent-14 using the PI-STKF scheme with a control horizon of $t_{H}=14\;s$ and temporal length scale of $15^{5}$ resulting in a noticeable belief change over time, as represented by the increasing predicted field variance (left column). The agent collects measurements from an spatio-temporal concentration field to compute an optimal path (|) based on the predicted STKF field variance. The next position of the agent’s trajectory (|) is obtained by applying the first value of the computed optimal control sequence. **Left:** Spatio-temporal concentration field **Center:** Predicted field mean **Right:** Predicted field variance.

In this section we examine the scenario of a spatio-temporal process. The field values are defined on the GMRF grid and interpolated between each other in order to obtain a continuous field. The temporal process at time instance t= 10 s, 30 s, 60 s, and 90 s is depicted in Figure 15(left column). We use the same parameters as in the PI-STKF simulation, see Table 3, except for the temporal length scale which we set to $15^{5}$. The reduction of temporal length scale let the agent’s belief variance increase over time if no measurement is obtained. Thus, the STKF is able to capture the evolution of the temporal process through the increase in uncertainty of the temporal process component. This is beneficial for coping with unknown stochastic components of the environmental process and hence potentially enables long-term autonomous monitoring scenarios.

Figure 15 illustrates the predicted field mean and variance of the STKF belief model, while the PI-STKF explores the spatio-temporal field. The spatio-temporal process at the agent’s initial position starts with a concentration value of approximately −1.5. After t=60s the agent has finished its initial clockwise exploration maneuver and passes the vicinity of its initial position again which concentration value has now changed to ca. 1.5. As depicted in Figure 9, the optimal control is computed by sampling potential path roll-outs and weighting them through a path probability that results from the conditioned variance as well as the control effort. As the variance of the field belief has noticeably increased during this maneuver, the obtained measurements lead to a process estimate which fairly represents the underlying original concentration field. Thereby, the increasing conditional field variance describes the loss of information in field regions which have not been visited by the agent recently. The time instance t=90s is a illustrative example on the controller exploration strategy: the planned trajectory first heads towards a local variance maximum and then points to the agent’s starting position at the beginning of the simulation. During the numerical experiment the computational time of the PI-STKF algorithm was on average 0.7 s and remained constant.

## 7. Conclusions

In this work, we outline the combination of Kernel-based belief models with stochastic optimal control while ensuring an upper bound on the computational load of the algorithm. First, a sequential fully Bayesian GMRF algorithm was extended to incorporate off-grid observations through shape-functions. Second, we showed that a spacetime Kalman filter belief model combined with the PI${}^{2}$ algorithm enables the exploration of spatio-temporal environmental fields. We demonstrated that the STKF algorithm can be extended by using shape functions to handle off-grid observations while remaining constant computational complexity. Extensive simulations were carried out to systematically identify bottlenecks in the exploration algorithm’s performance.

Future work will address the robustness of the PI-STKF algorithm while taking into account non-Gaussian measurement noise, uncertain localization, and uncertain hyperparameters. Furthermore, it is interesting to analyze the algorithms’ performance in an underwater experimental setup by using micro underwater robots as mobile sensor nodes. However, the design of meaningful experimental studies is a challenging task itself, as it requires reproducible environmental fields to provide the same scenario setup to all algorithms. Such a benchmark field can be realized by using, for instance, look-up tables of the field onboard the robots or spatial depending illumination of the fluid volumes which is then measured by the robots. Moreover, future studies will examine the influence of different field geometries (e.g., irregular grid shapes) on the exploration performance. These will also include varying control horizons and exploration noise. Another aspect is the analysis of different information theoretic criteria to redefine the state cost of the optimal control problem. Such future studies could evolve around the approximation of common information theoretic criteria for path planning such as mutual information [24] to increase exploration performance. In this context, further analysis of the belief models’ computational properties should be conducted. A combination of a GMRF which approximates the spatial process component while e.g., a Kalman Filter captures the temporal process component could result in a computationally efficient spatio-temporal GP model. Furthermore, the presented algorithms could extended to a multi-agent fleet which uses a decentralized belief representation rather than sharing one central belief model in order to reduce the dependence on restrictive underwater communication systems.

Finally, the proposed approach for synthesizing an autonomous field exploration algorithm shares similarities to the problem of safely learning dynamic models, such as in [28,29]. In the aforementioned works, the authors leverage GP models to safely explore the state-space of an unknown dynamic system, while the state can only changed continuously. We believe that the findings in one of those fields could be very fruitful for the other.

## Figures and Tables

**Table 1:** Computational complexity of the developed belief algorithms for two and three dimensions of the field belief with number of agents *N*, number of discrete time steps *k*, number of field grid values *n*, and dimension of the state space model *r*.

Belief Algorithm	2d	3d
GP Regression	$O({\left(Nk\right)}^{3})$	$O({\left(Nk\right)}^{3})$
Empirical GMRF Regression	$O\left(n^{3/2}\right)+O\left(Nk\right)$	$O\left(n^{2}\right)+O\left(Nk\right)$
Bayesian GMRF Regression	$O(Nn^{3/2})$	$O(Nn^{2})$
STKF	$O(rnN+N^{3})$	$O(rnN+N^{3})$

**Table 2:** Hyperparameter configurations of the individual algorithms and corresponding acronyms. The size of the belief field grid S is defined by the total number of vertices in *x*-direction being $n_{x}$. The second value inside the brackets depicts the total number of padding vertices in one dimension.

Acronym	Belief Algorithm		Process Type		Boundary Cond.
GMRF-1	Empirical GMRF		Matérn CAR(1)		Neumann
GMRF-2	Bayesian GMRF		Matérn CAR(1)		Neumann
GMRF-3	Bayesian GMRF		Matérn CAR(2)		Torus
STKF-1	STKF		Spat.: Matérn Cov. (ν=1) Temp.: Exp. Cov.		-
Acronym	$n_{x}\times n_{y}$	$\kappa^{2}$	τ	$\sigma_{f}$	*l*
GMRF-1	(80+10)×(40+10)	$10^{-5}$	1	-	-
GMRF-2	(80+10)×(40+10)	$10^{-4}$	0.5	-	-
GMRF-3	(40+20)×(20+20)	0.01	1	-	-
STKF-1	(40+0)×(20+0)	-	-	Spat.: 1.8 Temp.: 1	Spat.: 3.2 Temp.: $10^{7}$

**Table 3:** PI-STKF simulation parameters.

PI-Control Parameters	Symbol	Value
Control Horizon	$t_{H}$	4 s | 9 s | 14 s
Agent velocity	*v*	$0.5\;\frac{m}{s}$
Simulation time	-	150 s
Time step	Δt	1 s
Trajectory roll-outs	*K*	15
Control loop updates	$n_{\mathrm{updates}}$	10
Measurement variance	$\sigma_{y}^{2}$	0.3
Exploration noise	ε	π/10
Control cost	*R*	1

## Abbreviations

The following abbreviations are used in this manuscript:

GP	Gaussian process
GMRF	Gaussian Markov random field
STKF	Spacetime Kalman filter
SSM	State Space Model
CAR	Conditional auto-regressive
PI${}^{2}$	Policy improvement with path integrals
PI-GMRF	Combination of the GMRF belief model with the PI${}^{2}$ path planning algorithm
PI-STKF	Combination of the STKF belief model with the PI${}^{2}$ path planning algorithm
RMS	Root mean square
RL	Reinforcement learning
IQR	Inter-quarter range

## Appendix A. Sequential Update Rule for Continuous GMRF Algorithm

First, (20) is rewritten into a sum of vector products with $\Phi_{k,j}$ denoting the interpolation matrix of the *j*-th agent at discrete time step *k*, such that

$$\begin{array}{cc} \Lambda_{k|\theta } & =\Lambda_{k-1|\theta }+\frac{1}{\sigma_{y}^{2}}\Phi_{k}^{⊤}\Phi_{k}=\Lambda_{k-1|\theta }+\sum_{j=1}^{N}\frac{1}{\sigma_{y}^{2}}\Phi_{k,j}^{⊤}\Phi_{k,j}, \end{array}$$

(A1) $$\begin{array}{cc} & =\underset{\Lambda_{k-1,N|\theta }=\Lambda_{k|\theta }}{\underset{︸}{\underset{\Lambda_{k-1,1|\theta }}{\underset{︸}{\Lambda_{k-1|\theta }+\frac{1}{\sigma_{y}^{2}}\Phi_{k,1}^{⊤}\Phi_{k,1}}}+\frac{1}{\sigma_{y}^{2}}\Phi_{k,2}^{⊤}\Phi_{k,2}+\ldots +\frac{1}{\sigma_{y}^{2}}\Phi_{k,N}^{⊤}\Phi_{k,N}}}. \end{array}$$

By analyzes of the structure of (A1) an update rule is obtained that allows the sequential incorporation of new measurements as

(A2) $$\begin{array}{cc} \Sigma_{k-1,j|\theta } & =\Lambda_{k-1,j|\theta }^{-1}={\left(\Lambda_{k-1,j-1|\theta }+\frac{1}{\sigma_{y}^{2}}\Phi_{k,j}^{⊤}\Phi_{k,j}\right)}^{-1}. \end{array}$$

Applying the Sherman-Morrison formula on (A2) results in

(A3) $$\begin{array}{c} \Sigma_{k-1,j|\theta }=\Sigma_{k-1,j-1|\theta }-\frac{\Sigma_{k-1,j-1|\theta }\Phi_{k,j}^{⊤}\Phi_{k,j}\Sigma_{k-1,j-1|\theta }}{\sigma_{y}^{2}+\Phi_{k,j}\Sigma_{k-1,j-1|\theta }\Phi_{k,j}^{⊤}}. \end{array}$$

With (A1) and (A3), the conditional covariance matrix is obtained as

(A4) $$\begin{array}{cc} \Sigma_{k|\theta }= & \Sigma_{k-1|\theta }-\sum_{j=1}^{N}\frac{\Sigma_{k-1,j-1|\theta }\Phi_{k,j}^{⊤}\Phi_{k,j}\Sigma_{k-1,j-1|\theta }}{\sigma_{y}^{2}+\Phi_{k,j}\Sigma_{k-1,j-1|\theta }\Phi_{k,j}^{⊤}}=\Sigma_{k-1|\theta }-\sum_{j=1}^{N}\frac{h_{k,j}h_{k,j}^{⊤}}{\sigma_{y}^{2}+\Phi_{k,j}h_{k,j}}. \end{array}$$

Thus, the sequential update rule for the conditional variance can be written as

(A5) $$\mathrm{diag}\left(\Sigma_{k|\theta }\right)=\mathrm{diag}\left(\Sigma_{k-1|\theta }\right)-\sum_{j=1}^{N}\frac{h_{k,j}\circ h_{k,j}}{\sigma_{y}^{2}+\Phi_{k,j}h_{k,j}},\;\mathrm{with}$$

(A6) $$\begin{array}{cc} h_{k,j} & =\Sigma_{k-1,j|\theta }\Phi_{k,j}^{⊤}=\Lambda_{k-1,j|\theta }^{-1}\Phi_{k,j}^{⊤}, \end{array}$$

(A7) $$\begin{array}{cc} \Lambda_{k-1,j|\theta } & =\Lambda_{k-1|\theta }+\sum_{j=1}^{N}\frac{1}{\sigma_{y}^{2}}\Phi_{k,j}^{⊤}\Phi_{k,j}. \end{array}$$
